# Japan’s Demographic Dilemma: Navigating the Postpandemic Population Decline

**DOI:** 10.31662/jmaj.2024-0010

**Published:** 2024-06-03

**Authors:** Yudai Kaneda, Erika Yamashita, Uiri Kaneda, Tetsuya Tanimoto, Akihiko Ozaki

**Affiliations:** 1 School of Medicine, Hokkaido University, Sapporo, Japan; 2 Medical Governance Research Institute, Tokyo, Japan; 3 Faculty of Foreign Languages, Dokkyo University, Soka, Japan; 4 Department of Internal Medicine, Accessible Rail Medical Services Tetsuikai, Navitas Clinic Kawasaki, Kawasaki, Japan; 5 Department of Breast and Thyroid Surgery, Jyoban Hospital of Tokiwa Foundation, Iwaki, Japan

**Keywords:** COVID-19 Pandemic, Fertility Rates, Evidence-Based Policy Making (EBPM), Japan

## Abstract

In recent years, Japan has faced a significant demographic crisis, which was further exacerbated by the coronavirus disease 2019 (COVID-19) pandemic. By 2022, the country experienced a 1.5% decrease in population, which is in contrast to other G7 nations, and had the highest rate of excess mortality among Organization for Economic Co-operation and Development (OECD) countries. This crisis is mainly attributed to aging population, with Japan’s aging rate reaching 29.9%, the highest among its peer countries. The Japanese government, led by Prime Minister Fumio Kishida, has proposed policies aimed at addressing these challenges, focusing on increasing fertility rates. Despite these efforts, an evidence-based policymaking (EBPM) analysis reveals that the anticipated impact on fertility rates is marginal, with financial interventions estimated to yield only a slight population increase by 2060. Furthermore, the analysis highlighted the need for a more comprehensive approach, indicating that addressing societal issues such as gender norms and workplace culture might be crucial for a sustainable solution to Japan’s demographic challenges. This emphasizes the need for Japan to consider broader societal changes alongside fiscal policies to effectively combat its demographic decline.

In recent years, Japan has faced an acute demographic crisis exacerbated by the coronavirus disease 2019 (COVID-19) pandemic. By 2022, Japan’s population had decreased by 1.5% compared with the prepandemic year of 2019 ^(1)^. Such a decline is markedly in contrast to that of other G7 nations and the four East Asian regions, namely, Japan, China, South Korea, and Taiwan, with only Italy recording a similar population decrease of 1.16%. In addition, measures mainly focused on reducing human contact led to an increase of 166 deaths per 100,000 people compared with that in 2019, according to the Ministry of Health, Labour and Welfare’s vital statistics survey ^(2)^. The breakdown of this increase includes a 48-person increase in senility (49% increase), a 22-person increase in heart disease (14% increase), and a 13-person increase in aspiration pneumonia (41% increase) ^(2)^, cumulatively totaling to 111,000 excess deaths, which is six times the number of deaths due to COVID-19, and the highest rate of excess mortality among OECD countries, bringing to the fore the issue of an aging population ^(3)^. By 2022, Japan’s aging rate had reached 29.9%, making it not only the highest among the G7 countries and four East Asian regions but also significantly surpassing Italy, which had an aging rate of 24.1% ^(1)^. While factors such as immigration necessitate caution in making direct comparisons with Japan, the need for urgent measures in Japan is emphasized by the fact that advanced countries with significant aging populations, such as Germany at 22.4% and France at 21.7%, recorded population increases of 0.27% and 0.35%, respectively, during the pandemic ^(1)^.

The long-term demographic structure of a population is shaped by fertility and mortality rates as well as migration patterns. Among them, fertility rates are a pivotal factor in determining age composition, and in response to the challenges of an aging population, numerous countries have taken measures, such as immigration policies, to encourage higher birth rates ^(4)^. However, Japan has strong concerns regarding the potential impact of immigrants on the country’s social and cultural foundation, and because of linguistic barriers and a sense of ethnic homogeneity, its immigration policy has not progressed, remaining more restrictive compared with those in other advanced nations. Thus, Japanese Prime Minister Fumio Kishida has championed an ambitious and unique suite of measures to counteract the declining birth rates, which he describes as “different dimension” strategies.

Following the conceptualization of population decline as a relationship between economic decline and political change ^(5)^, government-sponsored coverage for children’s education can be a useful initiative, as it has been reported that population change is more influenced by educational factors than economic factors in Japan ^(6)^. However, the specifics of funding these policies have been postponed until the end of the year, creating uncertainty regarding the government’s capacity to realize its ambitious targets. Indeed, although Prime Minister Kishida has publicly committed to doubling the budget for child-related expenses by the early 2030s, a detailed plan to achieve this has not yet been described. Amid these developments, a government analysis has emerged, which critically evaluates the effectiveness of Japan’s strategies to address the declining birth rates through the lens of evidence-based policymaking (EBPM).

This analysis has revealed that even substantial interventions, such as increasing child allowances and other benefits, are projected to have only a marginal impact on fertility rates. Specifically, despite the allocation of resources equivalent to approximately 1% of GDP, or roughly 5 trillion yen, the anticipated increase in birth rate is marginal, with projections suggesting an increase of only 0.05-0.1 percentage points ^(7)^. In addition, it is estimated that by reducing the gender gap in domestic work hours to the OECD average, an additional increase in the fertility rate of around 0.1 percentage points could be achieved ^(7)^. However, the effect of these measures on population increase is estimated to be a cumulative total of 900,000-18,00,000 people from 2030 to 2060; furthermore, the fiscal expenditure committed by the Kishida administration to combat the declining birth rate is approximately 3.5 trillion yen per year, which is lower than the estimates, suggesting that the impact of these countermeasures on reversing demographic decline is extremely limited ^(7)^. This has led to a silent consensus within the government, where these sobering findings have been kept from public discourse, possibly to avoid political fallout.

The stark conclusion from the EBPM analysis is that Japan’s efforts to combat its demographic challenges may require a more radical and comprehensive approach. The analysis suggests that fiscal policy alone, without addressing deeper societal issues such as gender norms, workplace culture, and housing costs, is insufficient. In light of these insights, the administration of Prime Minister Kishida may need to consider not only the immediate impact of its policies on birth rates but also the broader societal changes, for example, introducing policies promoting flexible working hours and remote work options, with the aim of reducing the overtime culture and allowing more time for family and domestic duties, which is required to foster an environment conducive to family growth. Notably, such initiatives have been reported from the United States and Germany to lead to an increase in fertility intentions, particularly among highly educated women ^(8), (9)^. Although integrating strategies to reform workplace culture and improve male domestic participation into Japan’s unique societal fabric may be time-consuming, EBPM analysis suggests that these efforts could effectively boost population numbers similarly to financial measures ^(7)^, providing a sustainable and culturally transformative solution to Japan’s demographic crisis, and, thus, should be considered an urgent issue to address.

## Article Information

### Conflicts of Interest

Dr Ozaki reported personal fees from Medical Network Systems Inc. and Kyowa Kirin Co., Ltd., outside the submitted work. Dr Tanimoto reported personal fees from Medical Network Systems Inc., and Bionics Co., Ltd., outside the submitted work. No other disclosures were reported.

### Author Contributions

Conception and designing of the study: Yudai Kaneda and Tetsuya Tanimoto

Writing of the paper: Yudai Kaneda

Collection of data: Erika Yamashita

Critical revision of the paper: Uiri Kaneda and Akihiko Ozaki

All the authors read the final draft and approved submission
